# Twenty‐Four–Month Outcomes of Intravascular Ultrasound‐Guided Drug‐Coated Balloon Angioplasty for Femoropopliteal Artery Disease

**DOI:** 10.1161/JAHA.125.041564

**Published:** 2025-08-12

**Authors:** Jonghu Shin, Chul‐Min Ahn, Seung‐Jun Lee, Sang‐Hyup Lee, Yong‐Joon Lee, Byeong‐Keuk Kim, Myeong‐Ki Hong, Yangsoo Jang, Tae‐Hoon Kim, Ha‐Wook Park, Ji Yong Jang, Jae‐Hwan Lee, Jae‐Hyeong Park, Su Hong Kim, Eui Im, Sang‐ho Park, Donghoon Choi, Young‐Guk Ko, the IVUS‐DCB investigators

**Affiliations:** ^1^ Severance Cardiovascular Hospital Yonsei University College of Medicine Seoul Korea; ^2^ Division of Cardiology Hanil General Hospital Seoul Korea; ^3^ Division of Cardiology, Cardiovascular Center Bucheon Sejong Hospital Bucheon Korea; ^4^ Division of Cardiology Chungnam National University Sejong Hospital Sejong Korea; ^5^ Division of Cardiology Chungnam National University Hospital Daejeon Korea; ^6^ National Health Insurance Service Ilsan Hospital Goyang Korea; ^7^ Division of Cardiology Busan Veterans Hospital Busan Korea; ^8^ Division of Cardiology Yongin Severance Hospital Seoul Korea; ^9^ Cardiology Department Soonchunhyang University Cheonan Hospital Cheonan Korea

**Keywords:** drug‐coated balloon, femoropopliteal artery disease, imaging‐guided angioplasty, intravascular ultrasound, peripheral artery disease, Peripheral Vascular Disease, Revascularization, Treatment, Angiography, Ultrasound

## Abstract

**Background:**

The IVUS‐DCB (Intravascular Ultrasound‐Guided Drug‐Coated Balloon Angioplasty for Femoropopliteal Artery Disease) trial found that intravascular ultrasound (IVUS)–guided drug‐coated balloon (DCB) angioplasty was associated with superior 12‐month outcomes, compared with conventional angiography‐guided DCB angioplasty. However, the durability of these benefits remains uncertain. The aim of this study was to compare the 24‐month outcomes of IVUS‐guided versus angiography‐guided DCB angioplasty for femoropopliteal artery disease.

**Methods:**

This extended study analyzed a total of 237 patients who were previously randomized into IVUS‐guidance (n=119) or angiography‐guidance (n=118) groups in the original IVUS‐DCB trial. The 24‐month clinical efficacy outcomes included freedom from clinically driven target lesion revascularization, sustained clinical and hemodynamic improvements, and freedom from major amputation.

**Results:**

At 24‐month follow‐up, the IVUS‐guidance group exhibited significantly higher rates of freedom from clinically driven target lesion revascularization (87.4% versus 78.0%; hazard ratio, 0.46 [95% CI, 0.24–0.88]; *P*=0.02), sustained clinical improvement (82.4% versus 71.2%, *P*=0.02), and sustained hemodynamic improvement (74.8% versus 61.0%, *P*=0.01), compared with the angiography‐guidance group. No major amputations occurred in either group during the 24‐month follow‐up. There were no significant differences in safety outcomes between groups, including all‐cause death, cardiovascular death, and major bleeding.

**Conclusions:**

At 24‐month follow‐up, IVUS‐guided DCB angioplasty for femoropopliteal artery disease continued to demonstrate superior outcomes, compared with angiography‐guided DCB angioplasty, in terms of freedom from clinically driven target lesion revascularization, sustained clinical improvement, and sustained hemodynamic improvement.

## Graphical abstract

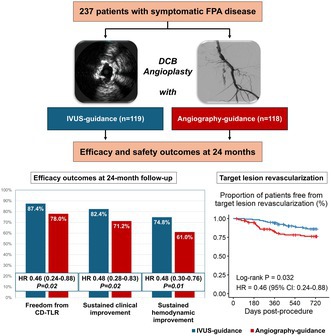

**Nonstandard Abbreviations and Acronyms**
CD‐TLR: clinically driven target lesion revascularization
DCB: drug‐coated balloon
EVT: endovascular therapy
FPA: femoropopliteal artery
IVUS: intravascular ultrasound
TASC: Trans‐Atlantic Inter‐Society Consensus

**Clinical Perspective**

**What Is New?**

- This study demonstrated that the procedural benefits of intravascular ultrasound–guided drug‐coated balloon angioplasty, previously observed at 12 months, were sustained over a 24‐month follow‐up period in patients with femoropopliteal artery disease.
- Intravascular ultrasound–guided drug‐coated balloon angioplasty yielded superior long‐term clinical outcomes compared with angiography‐guided procedures, including lower rates of target lesion revascularization and higher rates of sustained clinical and hemodynamic improvements, especially in patients with complex lesions (Trans‐Atlantic Inter‐Society Consensus II types C/D).

**What Are the Clinical Implications?**

- Intravascular ultrasound guidance may be considered to optimize long‐term outcomes and reduce reintervention rates in patients undergoing drug‐coated balloon angioplasty for femoropopliteal artery disease, particularly those with complex lesions.

The IVUS‐DCB (Intravascular Ultrasound–Guided Drug‐Coated Balloon Angioplasty for Femoropopliteal Artery Disease; NCT03517904) randomized clinical trial was designed to compare the clinical outcomes of intravascular ultrasound (IVUS)‐guided versus angiography‐guided drug‐coated balloon (DCB) angioplasty for the treatment of femoropopliteal artery (FPA) disease. The 1‐year results of that trial demonstrated significant clinical advantages of IVUS‐guided DCB angioplasty, particularly in terms of primary patency and reducing the need for reintervention, as evidenced by higher freedom from clinically driven target lesion revascularization (CD‐TLR), along with sustained clinical and hemodynamic improvements.^1^

Despite the promising 12‐month results after IVUS‐ DCB angioplasty, the durability of these benefits remains unclear. Previous studies have shown that primary patency after DCB treatment tends to decrease beyond 12 months, with increasing rates of target lesion revascularization or target vessel revascularization.^2^, ^3^, ^4^, ^5^ To date, no randomized trial has evaluated the mid‐ or long‐term effects of IVUS guidance during DCB angioplasty of FPA lesions beyond the 12‐month mark nor the sustainability of the 12‐month benefits during longer‐term follow‐up.

To address this issue, we conducted an extended follow‐up study to evaluate the clinical efficacy and safety outcomes beyond 12 months of patients enrolled in the IVUS‐DCB trial. This study aims to determine whether the benefits of IVUS guidance compared with angiography guidance observed at 12 months persist over the first 24 months after DCB angioplasty.

## METHODS

The data that support the findings of this study are available from the corresponding author upon reasonable request.

### Study Design and Data Collection

Detailed descriptions of the IVUS‐DCB trial design, inclusion and exclusion criteria, and 12‐month outcomes have been previously reported.^1^ The IVUS‐DCB trial was an investigator‐initiated, multicenter, randomized, single‐blinded study conducted at 7 centers across South Korea. It aimed to assess the clinical benefits of IVUS‐guided DCB angioplasty, compared with angiography‐guided DCB angioplasty, for the treatment of FPA disease.

Patients aged ≥19 years who were undergoing endovascular therapy (EVT) for symptomatic FPA disease (Rutherford categories 2–5) were eligible for enrollment in the IVUS‐DCB trial. Participants were randomly assigned in a 1:1 ratio to an IVUS‐guided group (n=119) or an angiography‐guided group (n=118). Detailed inclusion and exclusion criteria are shown in Table S1.^1^

The current study serves as an extended follow‐up investigation of the original IVUS‐DCB trial, with the period of observation extended to 24 months after the procedure. As the original study was designed with a follow‐up period of only 12 months, we obtained outcome data from 12 to 24 months after EVT by retrospective review of the electronic medical records from each of the 7 participating centers. Investigators from the original study were responsible for collecting this additional information.

The study protocol was approved by the institutional review board at each study site, and written informed consent was obtained from all patients for participation in the IVUS‐DCB trial. The requirement for written informed consent was waived for the current study because of its retrospective, observational nature, involving no further intervention. The study was conducted in accordance with the ethical principles outlined in the World Medical Association Declaration of Helsinki.

### Study End Points

The 24‐month clinical efficacy outcomes included freedom from CD‐TLR, sustained clinical and hemodynamic improvements, and freedom from major amputation. CD‐TLR was defined as reintervention because of significant target lesion stenosis of ≥50% within 5 mm proximal or distal to the original treatment segment, accompanied by symptom aggravation or a decrease in ankle–brachial index of ≥0.15.^6^, ^7^ Sustained clinical improvement was defined as an improved Rutherford category from baseline and freedom from major amputation or CD‐TLR.^8^ Sustained hemodynamic improvement was defined as an increase in ankle–brachial index by ≥0.15 from baseline and freedom from CD‐TLR.^9^ Major amputation was defined as any amputation of the target limb above the ankle.^10^ The 24‐month safety outcomes were all‐cause death, cardiovascular death, and major bleeding (defined according to the Thrombolysis in Myocardial Infarction criteria).^11^

Subgroup analyses of clinical outcomes were conducted on the basis of lesion complexity, as determined by the Trans‐Atlantic Inter‐Society Consensus (TASC) II classification. TASC II type C and D lesions were classified as complex lesions, whereas type A and B lesions were considered noncomplex lesions.^12^ Subgroup analyses of CD‐TLR were also conducted for other clinical and lesion characteristics, including diabetes status, lesion length, total occlusion, calcification, subintimal recanalization, and critical limb‐threatening ischemia (Rutherford categories 4 and 5).

### Statistical Analysis

All analyses for clinical outcomes were performed according to the intention‐to‐treat principle. Kaplan–Meier estimates were used to evaluate time‐to‐event data over the 24‐month follow‐up period, with between‐group comparisons conducted using the log‐rank test. Cox proportional hazards regression analysis was used to estimate hazard ratios (HRs) and 95% CIs for clinical outcomes, including subgroup analyses. Huber–White robust variance estimators were used for all Cox proportional hazards models. Interaction *P* values were calculated using the Wald test to evaluate potential differences in treatment effects across subgroups. Univariable and multivariable Cox proportional hazards regression analyses were performed to identify predictors of CD‐TLR and to estimate HRs and 95% CIs of potential predictors. Multivariable models were constructed using variables with *P* values <0.20 in the univariable analysis, with stepwise selection based on the Akaike information criterion (AIC). The study arm was forcibly included regardless of statistical significance. Multicollinearity was assessed using the variance inflation factor, and discriminative performance of the final model was evaluated using the area under the curve. The Holm–Bonferroni method was used to adjust for multiple comparisons in the analysis of efficacy outcomes. Continuous variables were reported as mean±SD or median (interquartile range) and were compared using Student's *t* test or the Mann–Whitney test, respectively. Categorical variables were presented as count and percentage, and groups were compared using the χ^2^ or Fisher's exact test, as appropriate. *P* values <0.05 were considered statistically significant. All analyses were performed using R statistical software version 4.4.1 (R Core Team).

## RESULTS

### Study Population

**Figure 1:**
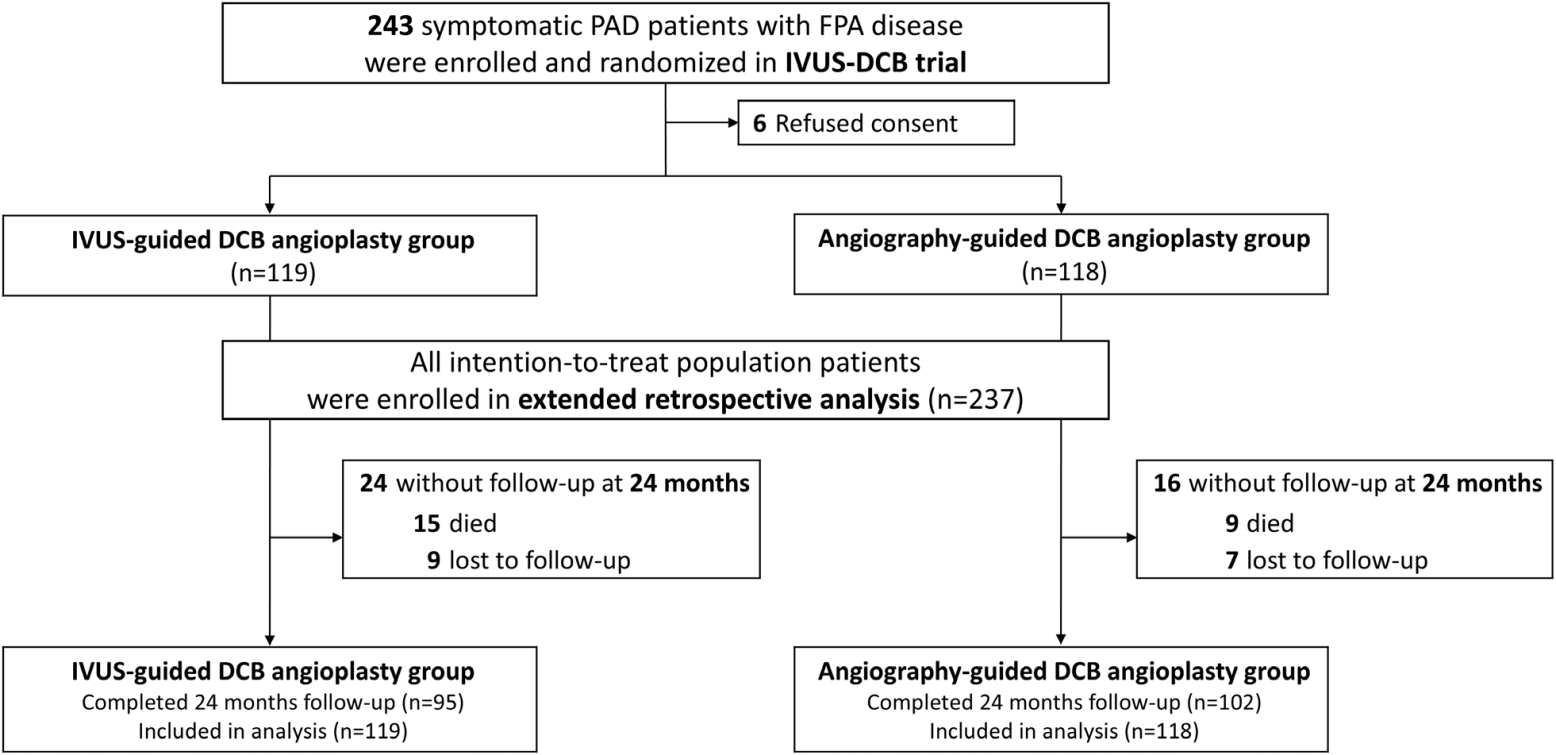
Flowchart of patients included in the current study. A total of 237 patients enrolled in the IVUS‐DCB trial were followed retrospectively over an extended period from 12 to 24 months after the procedure. All patients, including those who died or were lost to follow‐up, were included in the clinical outcome analyses. DCB indicates drug‐coated balloon; FPA, femoropopliteal artery; IVUS, intravascular ultrasound; IVUS‐DCB, Intravascular Ultrasound–Guided Drug‐Coated Balloon Angioplasty for Femoropopliteal Artery Disease; and PAD, peripheral artery disease.

The IVUS‐DCB trial enrolled 237 patients, randomized into an IVUS‐guidance group (n=119) and angiography‐guidance group (n=118). Over the 24‐month follow‐up period, 15 patients died and 9 were lost to follow‐up in the IVUS‐guidance group, whereas 9 patients died and 7 were lost to follow‐up in the angiography‐guidance group. A total of 197 patients, 95 (79.8%) in the IVUS‐guidance group and 102 (86.4%) in the angiography‐guidance group completed 24 months of follow‐up (Table S2). The current study included all patients, including those lost to follow‐up, as per the intention‐to‐treat design of the IVUS‐DCB trial (Figure 1).

As previously reported, the baseline characteristics of the IVUS‐guidance and angiography‐guidance groups were well matched, with similar demographics, comorbidities, and lesion characteristics.^1^ The patient and lesion baseline characteristics, procedural characteristics, and immediate postprocedural outcomes are presented in Tables 1 and 2.

**Table 1:** Baseline Patient and Angiographic Characteristics

Characteristics	IVUS guidance (n=119)	Angiography guidance (n=118)
Patient characteristics		
Age, y	69.0±9.1	70.2±8.6
Male sex	102 (85.7)	100 (84.7)
Hypertension	94 (78.0)	99 (83.8)
Diabetes	71 (59.7)	79 (67.5)
Dyslipidemia	84 (70.6)	86 (72.9)
Chronic kidney disease*	29 (24.4)	19 (16.1)
Current smoker	37 (31.1)	41 (34.7)
Critical limb‐threatening ischemia	30 (25.2)	32 (27.1)
Preprocedural ankle–brachial index	0.64±0.21	0.63±0.21
Angiographic characteristics		
Lesion length, mm	200.6 (120.0–270.9)	220.7 (124.7–286.7)
Total occlusion	78 (66.7)	68 (58.1)
TASC type		
A or B	39 (32.8)	40 (33.9)
C or D	80 (67.2)	78 (66.1)
Popliteal involvement	11 (9.2)	10 (8.5)

Variables are described as mean±SD, median (interquartile range), or number (%). IVUS indicates intravascular ultrasound; and TASC, Trans‐Atlantic Inter‐Society Consensus.

* Estimated glomerular filtration rate <60 mL/min per 1.73 m^2^ body surface area.

**Table 2:** Procedural Characteristics and Immediate Procedural Outcomes

Procedural characteristics and immediate outcomes	IVUS guidance (n=119)	Angiography guidance (n=118)	*P* value
Procedural characteristics			
Maximal DCB diameter, mm	5.8±0.7	5.8±0.7	0.95
Mean DCB diameter, mm	5.4±0.6	5.4±0.6	0.92
Preballoon diameter, mm	5.0±0.9	4.5±1.1	<0.001
Preballoon maximal pressure, mm Hg	11.8±3.6	8.9±2.7	<0.001
Use of atherectomy device	41 (35.0)	38 (32.5)	0.78
Adjuvant postdilatation	31 (26.1)	16 (13.6)	0.03
Maximal postballoon pressure, mm Hg	13.7±2.9	9.6±4.0	0.001
Bailout stenting	24 (20.5)	17 (14.5)	0.30
Postprocedural minimal lumen diameter, mm	3.90±0.59	3.71±0.73	0.03
Postprocedural diameter stenosis, %	21.5±12.0	25.4±13.3	0.02
Immediate procedural outcomes			
Technical success*	91 (76.5)	72 (61.0)	0.02
Procedural success†	88 (73.9)	71 (60.2)	0.03
Dissection type	70 (59.8)	68 (58.1)	0.67
A to C	63 (90.0)	62 (91.2)	…
D or E	7 (10.0)	6 (8.8)	…
Postprocedure ankle–brachial index‡	0.99±0.13	0.93±0.15	0.001

Variables are described as mean±SD or number (%). DCB indicates drug‐coated balloon; and IVUS, intravascular ultrasound.

* Residual stenosis <30% without flow compromise.

^†^ Achievement of technical success without any acute procedure‐related complications.

^‡^ Measured within 48 h after the index procedure.

### Clinical Efficacy Outcomes Through 24‐Month Follow‐Up

**Figure 2:**
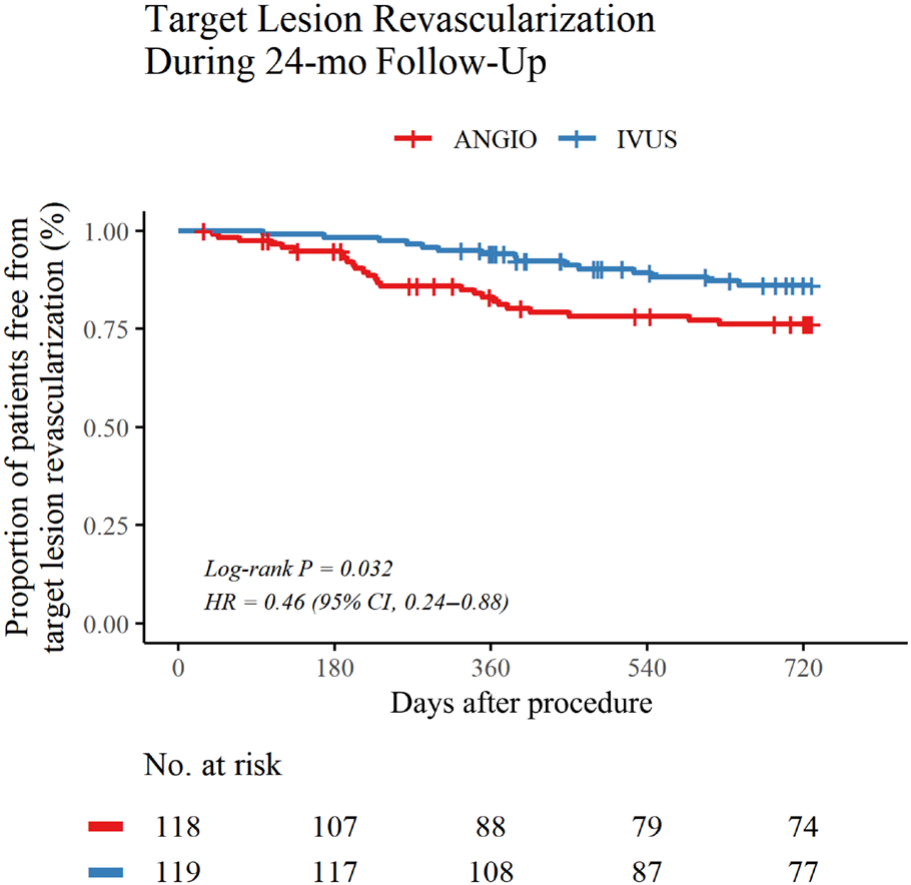
Kaplan–Meier curves for CD‐TLR in the first 24 months after the procedure. Kaplan–Meier estimates showed significantly higher freedom from CD‐TLR in the IVUS‐guidance group than in the angiography‐guidance group over the 24‐month follow‐up period (log‐rank *P=*0.032). The number of patients at risk of CD‐TLR in each group at specific time intervals is shown below the curves. Hazard ratios were adjusted for lesion length (cutoff value of 150 mm). ANGIO indicates angiography‐guided drug‐coated balloon angioplasty group; CD‐TLR, clinically driven target lesion revascularization; HR, hazard ratio; IVUS, intravascular ultrasound; and TASC, Trans‐Atlantic Inter‐Society Consensus.

The clinical efficacy outcomes during 24‐month follow‐up are summarized in Table 3, and Kaplan–Meier curves are presented in Figure 2. CD‐TLR occurred in 15 patients (12.6%) in the IVUS‐guidance group and 26 patients (22.0%) in the angiography‐guidance group during the first 24 months after the procedure, resulting in a statistically significant higher rate of freedom from CD‐TLR in the IVUS‐guidance group (87.4% versus 78.0%; HR, 0.46 [95% CI, 0.24–0.88]; *P=*0.02). The IVUS‐guidance group also had significantly higher rates of sustained clinical improvement (82.4% versus 71.2%; HR, 0.48 [95% CI, 0.28–0.83]; *P=*0.02) and sustained hemodynamic improvement (74.8% versus 61.0%; HR, 0.48 [95% CI, 0.30–0.76]; *P=*0.01) over the 24‐month follow‐up period, compared with the angiography‐guidance group. No major amputations occurred in either group.

**Table 3:** Efficacy and Safety Outcomes at 24 Months After Drug‐Coated Balloon Angioplasty

Variable	IVUS guidance (n=119)	Angiography guidance (n=118)	Hazard ratio* (95% CI)	*P* value
Efficacy outcomes				
Freedom from CD‐TLR	87.4 (104/119)	92/118 (78.0)	0.46 (0.24–0.88)	0.02
Time to first CD‐TLR, d†	379.7±160.5	261.7±151.0	…	…
Sustained clinical improvement‡	82.4 (98/119)	84/118 (71.2)	0.48 (0.28–0.83)	0.02

Sustained hemodynamic improvement§	74.8 (89/119)	72/118 (61.0)	0.48 (0.30–0.76)	0.01
Major amputation of target limb	0 (0/119)	0/118 (0)	…	…
Safety outcomes				
All‐cause death	12.6 (15/119)	9/118 (7.6)	1.65 (0.72–3.76)	0.24
Cardiovascular death	2.5 (3/119)	3/118 (2.5)	0.99 (0.20–5.01)	0.99
Major bleeding	4.2 (5/119)	4.2 (5/118)	1.03 (0.30–3.53)	0.97

Mean±SD or n/N (percentage). CD‐TLR indicates clinically driven target lesion revascularization; DCB, drug‐coated balloon; and IVUS, intravascular ultrasound.

* Hazard ratios are for IVUS‐guided DCB angioplasty vs angiography‐guided DCB angioplasty, calculated using Cox proportional hazards model adjusted for lesion length (cutoff value of 150 mm).

^†^ The IVUS‐guided group had significantly longer time to event compared with the angiography‐guided group (rate ratio, 1.45 [95% CI, 1.06–2.01]; *P*=0.02), assessed by negative binomial regression analysis.

^‡^ Increase in Rutherford class from baseline and freedom from target limb major amputation or CD‐TLR.

^§^ Increase in ankle–brachial index ≥0.15 from baseline and freedom from CD‐TLR.

### Safety Outcomes Through 24 Months

The safety outcomes during the 24‐month follow‐up are summarized in Table 3. There were no significant differences between the IVUS‐guidance and angiography‐guidance groups with respect to rates of all‐cause death (12.6% versus 7.6%, respectively; *P=*0.24) or cardiovascular death (2.5% versus 2.5%, *P=*0.99). No procedure‐ or device‐related deaths occurred in either group. Rates of major bleeding events also did not differ between groups (4.2% versus 4.2%, *P=*0.97).

### Subgroup Analyses

**Figure 3:**
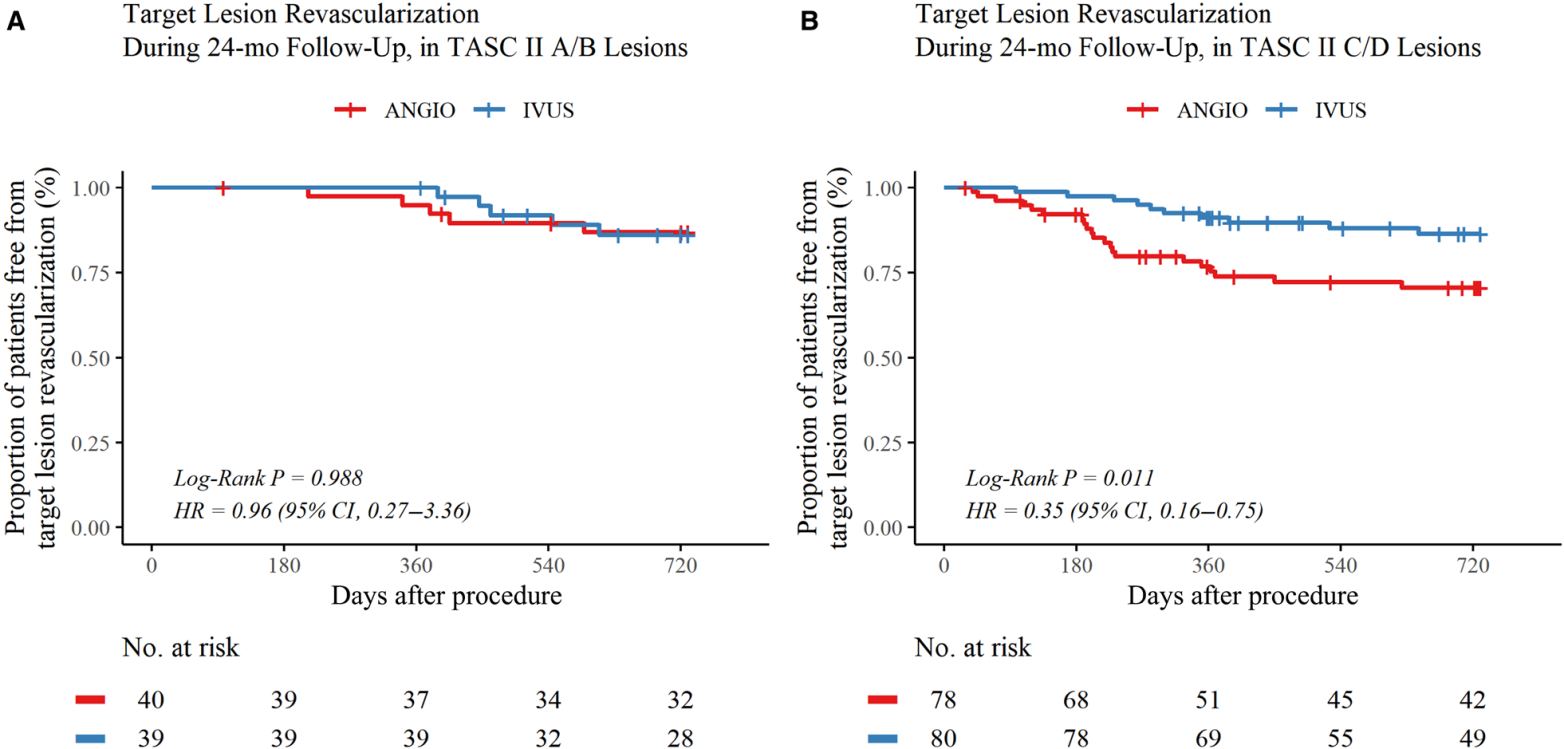
Kaplan–Meier curves for CD‐TLR in the first 24 months after the procedure, stratified according to lesion complexity. Kaplan–Meier estimates showed no significant difference in rates of CD‐TLR between the IVUS‐guidance and angiography‐guidance groups in patients with noncomplex lesions (**A**) (log‐rank *P*=0.99). Significant differences were observed between groups in patients with complex lesions (**B**) (log‐rank *P*=0.011). The number of patients at risk of CD‐TLR in each group at specific time intervals is shown below the curves. Hazard ratios were adjusted for lesion length (cutoff value of 150 mm). ANGIO indicates angiography‐guided drug‐coated balloon angioplasty group; CD‐TLR, clinically driven target lesion revascularization; HR, hazard ratio; IVUS, intravascular ultrasound; and TASC, Trans‐Atlantic Inter‐Society Consensus.

**Figure 4:**
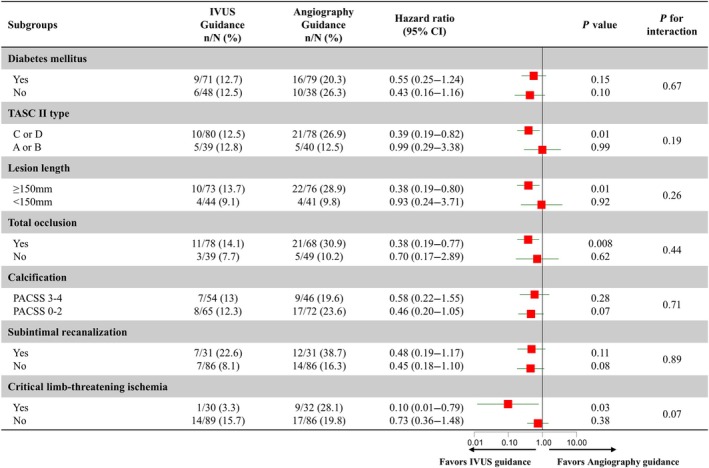
Subgroup analyses of CD‐TLR in the first 24 months after the procedure according to patient and lesion characteristics. Forest plots show the results of subgroup analyses of 24‐month CD‐TLR according to various patient and lesion characteristics. IVUS guidance was associated with favorable outcomes in patients with high‐risk lesions, including long lesions, total occlusions, and lesions associated with critical limb‐threatening ischemia (*P<*0.05). CD‐TLR indicates clinically driven target lesion revascularization; IVUS, intravascular ultrasound; PACSS, Peripheral Arterial Calcium Scoring System; and TASC, Trans‐Atlantic Inter‐Society Consensus.

Rates of freedom from CD‐TLR did not differ significantly between IVUS‐guidance and angiography‐guidance groups in patients with noncomplex (TASC II type A/B) lesions (Figure 3). By contrast, IVUS guidance was associated with a higher rate of freedom from CD‐TLR than angiography guidance in patients with complex (TASC II type C/D) lesions (87.5% versus 73.1%; HR, 0.35 [95% CI, 0.16–0.75]; *P=*0.01). Rates of sustained clinical and hemodynamic improvements were also higher in the IVUS‐guidance group than in the angiography‐guidance group in patients with complex lesions, as shown in Figure S1. Subgroup analyses of CD‐TLR based on clinical and other lesion characteristics are shown in Figure 4. Compared with angiography guidance, IVUS guidance was associated with a lower rate of CD‐TLR in lesions with several high‐risk characteristics, including long lesions, total occlusions, and lesions causing critical limb‐threatening ischemia (all *P<*0.05; Figure 4). IVUS guidance did not significantly affect the rate of CD‐TLR in lesions without high‐risk characteristics.

### Independent Predictors of Target Lesion Revascularization

Table 4 presents potential predictors of 24‐month CD‐TLR. Among candidate variables with *P* values <0.20 in the univariable analysis, sex, end‐stage renal disease with hemodialysis, total occlusion, popliteal artery involvement, lesion length ≥200 mm, and IVUS guidance were selected through stepwise selection based on minimal Akaike information criterion. The final model had an area under the curve of 0.73, and all included variables had variance inflation factor values <2, indicating no significant multicollinearity. IVUS guidance was identified as an independent predictor of a lower hazard of CD‐TLR (HR, 0.37 [95% CI, 0.19–0.72]; *P*=0.003), whereas total occlusion (HR, 2.34 [95% CI, 1.02–5.36]; *P*=0.045), popliteal artery involvement (HR, 2.62 [95% CI, 1.14–6.02]; *P*=0.02), and lesion length ≥200 mm (HR, 2.74 [95% CI, 1.24–6.02]; *P*=0.01) were predictors of increased hazard of CD‐TLR.

**Table 4:** Predictors of Target Lesion Revascularization During 24‐Month Follow‐Up After the Procedure

Variables	Univariable		Multivariable	
	HR (95% CI)	*P* value	HR (95% CI)	*P* value
Age, y	0.97 (0.94–1.01)	0.18		
Male sex	2.18 (0.66–7.19)	0.20	2.46 (0.69–8.96)	0.16
Body mass index, kg/m^2^	0.97 (0.87–1.08)	0.59		
Hypertension	0.90 (0.41–1.97)	0.80		
Diabetes	0.89 (0.47–1.67)	0.72		
Dyslipidemia	0.82 (0.42–1.57)	0.54		
Chronic kidney disease	1.15 (0.55–2.38)	0.72		
End‐stage renal disease treated with hemodialysis	2.06 (0.87–4.90)	0.10	2.19 (0.70–6.91)	0.18
Current smoker	1.47 (0.78–2.77)	0.23		
Coronary artery disease	0.61 (0.29–1.27)	0.19		
Prior peripheral revascularization	1.36 (0.62–2.95)	0.44		
Critical limb‐threatening ischemia	1.07 (0.52–2.21)	0.85		
Total occlusion	2.68 (1.26–5.73)	0.01	2.34 (1.02–5.36)	0.045
Severe calcification (PACSS score of 4)	0.66 (0.32–1.35)	0.25		
Poor distal runoff*	1.20 (0.63–2.31)	0.58		
Popliteal artery involvement	2.12 (0.97–4.64)	0.06	2.62 (1.14–6.02)	0.02
Lesion length ≥200 mm	3.48 (1.71–7.12)	0.001	2.74 (1.24–6.02)	0.01
IVUS guidance	0.51 (0.27–0.95)	0.03	0.37 (0.19–0.72)	0.003

HR indicates hazard ratio; IVUS, intravascular ultrasound; and PACSS, Peripheral Arterial Calcium Scoring System.

* Only 1 or no patent runoff vessels.

## DISCUSSION

This retrospective, multicenter, extended follow‐up study of the IVUS‐DCB trial demonstrated that IVUS‐guided DCB angioplasty provided superior 24‐month clinical outcomes, compared with angiography‐guided DCB, when used for EVT of FPA disease. At 24 months, the IVUS‐guidance group had a significantly lower rate of CD‐TLR, as well as higher rates of sustained clinical and hemodynamic improvements, compared with the angiography‐guidance group, with no significant differences in safety outcomes between groups.

In the initial IVUS‐DCB trial, IVUS guidance during DCB angioplasty for FPA disease was associated with improved immediate procedural outcomes, including a higher technical success rate and better postprocedural ankle–brachial index, which contributed to superior 12‐month clinical outcomes, including enhanced primary patency, improved freedom from CD‐TLR, and sustained clinical and hemodynamic improvements.^1^ The use of IVUS allows precise measurement of vessel and lumen diameters, facilitating the selection of appropriately sized devices and optimizing lesion dilatation during EVT. It has been reported that IVUS tends to measure reference vessel diameters approximately 1 mm larger than angiography.^13^

This explains why more aggressive pre‐ and postdilation with larger balloons and higher inflation pressures were performed in the IVUS‐guided group compared with the angiography‐guided group. The higher technical success rate in the IVUS‐guided group was attributed to achieving a larger lumen diameter through effective pre‐ and post‐DCB lesion optimization.

Few studies have evaluated mid‐ or long‐term clinical outcomes after IVUS‐guided DCB angioplasty for FPA disease. The results of our current study demonstrate that the benefits of IVUS guidance are sustained beyond 12 months. Notably, these effects were most pronounced in complex lesions (TASC II types C/D), suggesting that IVUS guidance may offer durable and sustained advantages for challenging lesions, extending the 12‐month outcomes demonstrated in a recent subgroup analysis of the IVUS‐DCB trial.^12^

Although previous studies have compared IVUS‐guided and angiography‐guided angioplasty for peripheral artery lesions, they involved a variety of lesion locations and endovascular devices (plain balloons, bare‐metal stents, DCBs, drug‐eluting stents, and atherectomy devices), resulting in conflicting results.^14^, ^15^, ^16^, ^17^, ^18^, ^19^, ^20^, ^21^, ^22^, ^23^ Several large retrospective cohort studies have reported inconsistent outcomes for IVUS‐guided EVT for lower extremity peripheral artery disease.^14^, ^15^, ^16^ Brahmandam et al found that IVUS‐guided EVT was associated with improved primary patency,^14^ while Divakaran et al reported that IVUS use during EVT was linked to lower risks of major adverse limb events, acute limb ischemia, and major amputation.^15^ By contrast, Setogawa et al observed higher risks of reintervention and readmission with IVUS guidance, despite lower risks of bypass surgery or stent grafting.^16^ These discrepancies are difficult to compare directly, as the studies included diverse lesion locations and EVT devices. Furthermore, 2 meta‐analyses found no statistically significant differences in primary patency or reintervention rates between IVUS and angiography guidance during EVT, despite notable reductions in procedure‐related complications with IVUS use.^18^, ^19^ In a randomized controlled trial by Allan et al, IVUS guidance during EVT for FPA lesions with various devices significantly reduced restenosis rates within 12 months, particularly in cases involving DCBs, consistent with the findings of the IVUS‐DCB trial.^20^ Nevertheless, CD‐TLR rates did not differ significantly between IVUS‐guidance and angiography‐guidance groups in the Allan et al. study. Use of IVUS during EVT with self‐expanding bare‐metal stents has been demonstrated to reduce reintervention rates and improve primary patency^21^, ^22^; however, IVUS guidance during EVT with drug‐eluting stents did not reduce restenosis rates and was associated with an increased incidence of aneurysmal degeneration.^23^

To date, the specific types of lesions that may derive greater benefit from IVUS guidance during EVT with DCBs remain unclear. Previously, Iida et al reported that IVUS‐guided EVT using plain balloons and bare‐metal stents significantly improved long‐term patency in TASC II type A to C FPA lesions.^21^ However, this benefit was not observed in TASC II type D lesions. By contrast, Brahmandam et al demonstrated that IVUS guidance was particularly effective for EVT in patients with critical limb‐threatening ischemia and TASC II type C and D lesions.^14^ Similarly, we found that the clinical benefits of IVUS guidance were most pronounced in complex lesions (TASC II types C/D). In a recent subgroup analysis of the IVUS‐DCB trial, IVUS guidance resulted in greater postprocedural minimal lumen diameter, higher technical success rates, and improved postprocedure ankle–brachial index. In contrast, no significant differences were observed between the groups in technical success or postprocedural minimal lumen diameter for noncomplex FPA. These findings suggest that angiography alone may be inadequate for optimizing procedural outcomes in complex lesions.^12^ Our finding suggests that these benefits of IVUS guidance were sustained during the 24‐month follow‐up, highlighting the potential of IVUS guidance for achieving optimal outcomes in challenging lesions.

Multivariable Cox regression analyses revealed that IVUS guidance was an independent predictor of a lower hazard of CD‐TLR within 24 months following DCB angioplasty, whereas total occlusion, popliteal artery involvement, and longer lesion length were predictors of increased hazard of CD‐TLR. These findings are consistent with the results of the original IVUS‐DCB trial, as well as other previous studies.^1^, ^24^, ^25^

### Study Limitations

This study has some limitations. First, data collection for the extended follow‐up period from 12 to 24 months was performed retrospectively and relied mainly on information obtained from electronic medical records. In addition, clinical outcomes were not adjudicated in a blinded manner, which may have introduced bias. Although efforts were made to ensure data accuracy, the retrospective nature of this data collection is associated with an inherent risk of missing or incomplete data, which could impact the robustness of our results. Second, unlike the original IVUS‐DCB 12‐month trial, we did not evaluate primary patency as a primary outcome in this extended follow‐up study because the original study protocol was designed as a 12‐month follow‐up clinical trial. Therefore, the 24‐month clinical outcome data had to be collected retrospectively. Unfortunately, imaging studies were not routinely performed at the 24‐month follow‐up, which limited the assessment of primary patency at that time point. Third, this study was conducted exclusively in South Korea, which may limit the generalizability of our findings to other populations, ethnic groups, or health care systems. Finally, the relatively small sample size may reduce the statistical power to detect significant differences between subgroups. In particular, the nonsignificant benefit of IVUS in noncomplex FPA may be attributed to small sample size (n=79). Therefore, these findings should be considered exploratory and serve as a basis for future studies with larger populations.

## CONCLUSIONS

In patients with FPA disease, IVUS‐guided DCB angioplasty was associated with significantly better clinical outcomes during 24 months of follow‐up compared with angiography‐guided DCB angioplasty. These benefits included higher rates of freedom from CD‐TLR, presence of sustained clinical improvement, and presence of sustained hemodynamic improvement. These findings highlight the sustained long‐term advantages of IVUS guidance in optimizing lesion treatment and improving outcomes for patients with symptomatic FPA disease.

## Sources of Funding

This study was funded by Medtronic Inc. and Korea United Pharmaceutical and supported by the Cardiovascular Research Center, Seoul, Korea.

## Disclosures

Drs Young‐Guk Ko and Donghoon Choi have received research grants from Medtronic, Korea United Pharm, Cook Medical, Boston Scientific, Otsuka Korea, Dong‐A ST, Samjin Pharm, and Cordis. All other authors declare no conflicts of interest.

## Supporting information

Tables S1–S2
Figure S1
