# Supplementary material for: Twenty‐Four–Month Outcomes of Intravascular Ultrasound‐Guided Drug‐Coated Balloon Angioplasty for Femoropopliteal Artery Disease
Source: J Am Heart Assoc. 2025 Aug 12;14(16):e041564. doi: 10.1161/JAHA.125.041564 (PMC12748071; doi:10.1161/JAHA.125.041564)
Supplement: Supplementary file 1 — Tables S1–S2 Figure S1 [file JAH3-14-e041564-s001.pdf]

SUPPLEMENTAL MATERIAL

Table S1. Inclusion and exclusion criteria.

Inclusion Criteria (all criteria below should be met)
1. Age 19 or older
2. Symptomatic peripheral artery disease<br>· Moderate or severe claudication (Rutherford classification grade 2 or 3) or<br>· Critical limb threatening ischemia (Rutherford classification grade 4 or 5)
3. Stenosis of more than 50% of the femoropopliteal artery
4. Ankle-brachial index <0.9
**Exclusion Criteria**
1. Acute severe lower extremity ischemia
2. Severe critical limb ischemia (Rutherford classification 6)
3. Known hypersensitivity or contraindications to the following medicines: heparin, aspirin, clopidogrel, cilostazol, or contrast media
4. Patients who need to take oral anticoagulants such as warfarin or NOAC
5. Age > 85 years old
6. Severe liver function abnormality (more than 3 times the normal reference value)
7. Significant leukopenia, neutropenia, thrombocytopenia, anemia or known hemorrhagic tendency
8. Left ventricular ejection fraction <40% or clinically evident congestive heart failure
9. Pregnant women or women of childbearing age
10. Patients with an expected life expectancy of less than 1 year due to comorbidities
11. Patients with a history of bypass surgery or stent insertion involving the target femoropopliteal artery
12. When there is a lesion with >50% stenosis above the target femoropopliteal artery lesion (iliac artery or proximal femoral artery) without revascularization.<br>■ The subject can be enrolled if the inflow lesion is performed concomitantly with the target femoropopliteal artery lesion.)

NOAC denotes Non-vitamin K antagonist oral anticoagulants

Table S2. Baseline characteristics of patients who completed 24-month follow-up versus those who died or were lost to follow-up.

Characteristics	Died or lost to follow-up		Completed follow-up	
	**IVUS Guidance (n=24)**	**Angiography Guidance (n=16)**	**IVUS Guidance (n=95)**	**Angiography Guidance (n=102)**
Patient characteristics				
Age, years	70.8±10.3	75.8±5.7	68.6±8.7	69.3±8.6
Male	19 (79.2)	12 (75.0)	83 (87.4)	88 (86.3)
Hypertension	18 (75.0)	14 (87.5)	76 (80.0)	84 (83.2)
Diabetes mellitus	16 (66.7)	12 (75.0)	55 (57.9)	67 (66.3)
Dyslipidemia	16 (66.7)	12 (75.0)	68 (71.6)	74 (72.5)
Chronic kidney disease*	10 (41.7)	5 (31.2)	19 (20.0)	14 (13.7)
Current smoker	6 (25.0)	6 (37.5)	31 (32.6)	26 (25.5)
Critical limb-threatening ischemia	14 (58.3)	7 (43.8)	16 (16.8)	25 (24.5)
Pre-procedural ABI	0.62±0.22	0.56±0.19	0.64±0.21	0.64±0.22
Angiographic characteristics				
Lesion length, mm	240.8 [205.3; 256.5]	255.6 [187.7; 284.4]	189.6 [112.3; 288.1]	203.3 [123.1; 286.7]
Total occlusion	16 (69.6)	9 (56.2)	62 (66.0)	59 (58.4)
TASC type				
A or B	6 (25.0)	3 (18.8)	33 (34.7)	37 (36.3)
C or D	18 (75.0)	13 (81.2)	62 (65.3)	65 (63.7)
Popliteal involvement	2 (8.3)	1 (6.2)	9 (9.5)	9 (8.9)

Variables are described as mean±standard deviation or median [interquartile range] or number (%).
Abbreviations: ABI, ankle-brachial index; IVUS, intravascular ultrasound; TASC, Trans-Atlantic Inter-Society Consensus.
*Estimated glomerular filtration rate < 60 mL/min/1.73 m^2 body surface area.

Figure S1. Efficacy outcomes at 24 months stratified by lesion complexity.

Efficacy outcomes	IVUS Guidance n/N (%)	Angiography Guidance n/N (%)	Hazard ratio* (95% CI)	P value	P for interaction
**Freedom from CD-TLR**					
TASC II C/D	70/80 (87.5%)	57/78 (73.1%)	0.35 (0.16-0.75)	0.01	0.15
TASC II A/B	34/39 (87.2%)	35/40 (87.5%)	0.96 (0.27-3.36)	0.95	
**Sustained clinical improvement†**					
TASC II C/D	66/80 (81.2%)	53/78 (67.9%)	0.43 (0.23-0.82)	0.01	0.53
TASC II A/B	33/39 (84.6%)	31/40 (77.5%)	0.64 (0.23-1.77)	0.39	
**Sustained hemodynamic improvement‡**					
TASC II C/D	60/80 (75.0%)	45/78 (57.7%)	0.43 (0.25-0.75)	0.003	0.39
TASC II A/B	29/39 (74.4%)	27/40 (67.5%)	0.61 (0.27-1.35)	0.22	

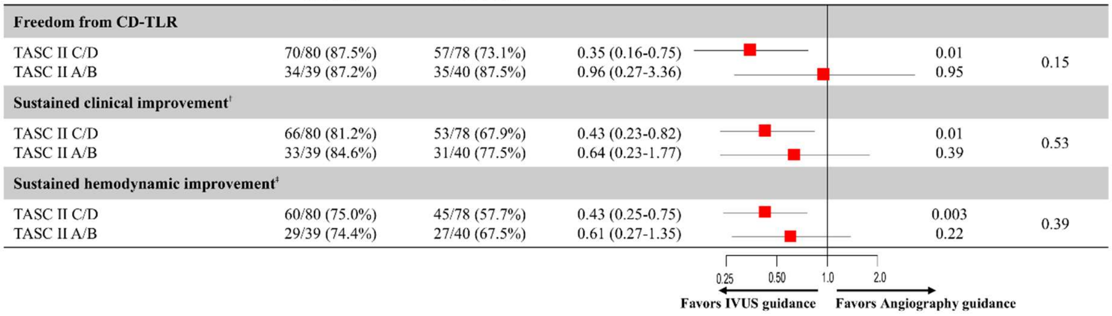

Values are described as mean±standard deviation or percentage (n/N).

Abbreviations: IVUS, intravascular ultrasound; CI, confidence interval; DCB, drug-coated balloon; CD-TLR, clinically driven target lesion revascularization; HR, hazard ratio; TASC, Trans- Atlantic Inter-Society Consensus.
*Hazard ratios are for IVUS-guided DCB angioplasty versus angiography-guided DCB angioplasty, calculated using Cox proportional hazards model adjusted for lesion length (cut-off value of 150 mm).

†Increase in Rutherford class from baseline and freedom from target limb amputation or CD-TLR.

‡Increase in the ankle-brachial index ≥ 0.15 from baseline and freedom from CD-TLR.
